# Supplementary material for: Team Assembly Mechanisms and the Knowledge Produced in the Mexico's National Institute of Geriatrics: A Network Analysis and Agent-Based Modeling Approach
Source: Scientifica (Cairo). 2019 Mar 3;2019:9127657. doi: 10.1155/2019/9127657 (PMC6421728; doi:10.1155/2019/9127657)

Bimodal networks of papers and authors | **Collaboration networks**

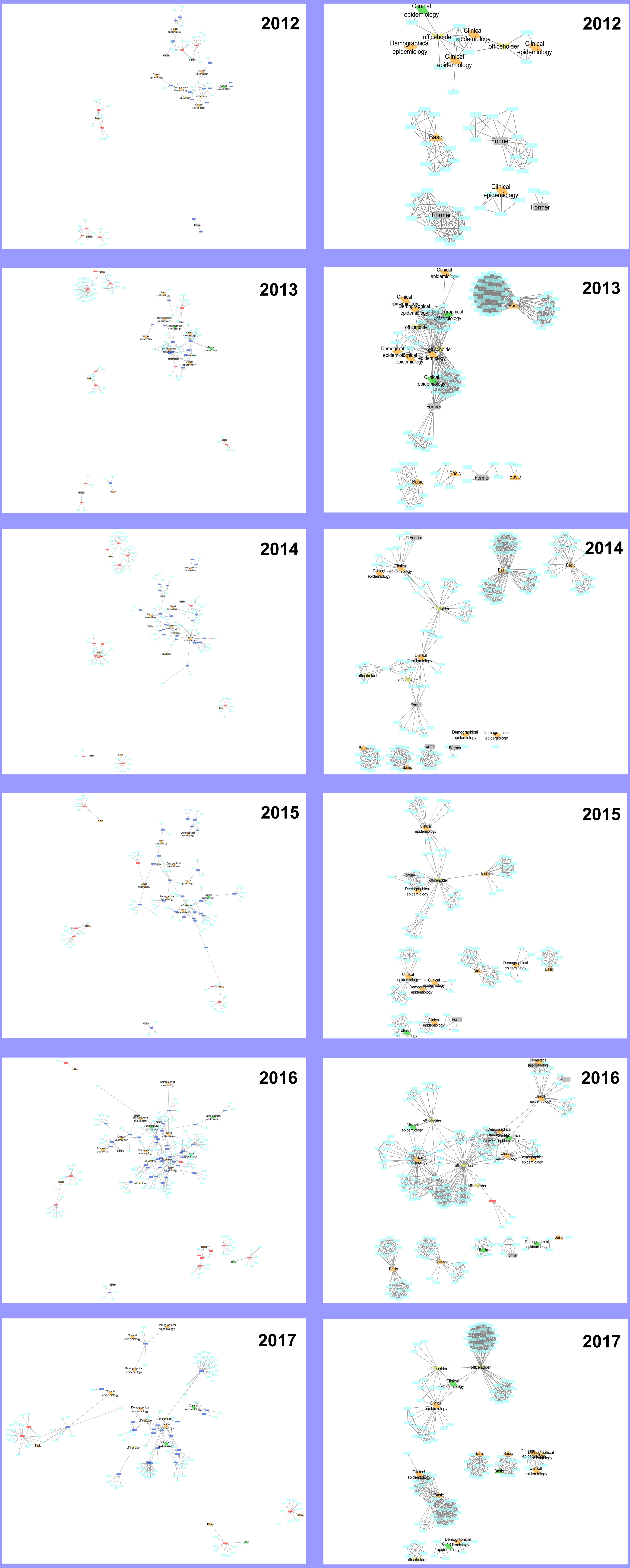

Supplement: Supplementary Materials — Collaboration network model of INGER authors sliced by years (2012–2017) is shown. The authors were equally colored as in Figure 2. [file 9127657.f1.pdf]
